# A Chemical Genetic Screen for Modulators of Asymmetrical 2,2′-Dimeric Naphthoquinones Cytotoxicity in Yeast

**DOI:** 10.1371/journal.pone.0010846

**Published:** 2010-05-26

**Authors:** Ashkan Emadi, Ashley E. Ross, Kathleen M. Cowan, Yolanda M. Fortenberry, Milena Vuica-Ross

**Affiliations:** 1 Department of Internal Medicine and Oncology, Johns Hopkins School of Medicine, Baltimore, Maryland, United States of America; 2 Department of Urology, Johns Hopkins School of Medicine, Baltimore, Maryland, United States of America; 3 Department of Pathology, Johns Hopkins School of Medicine, Baltimore, Maryland, United States of America; 4 Department of Pediatrics, Johns Hopkins School of Medicine, Baltimore, Maryland, United States of America; Baylor College of Medicine, United States of America

## Abstract

**Background:**

Dimeric naphthoquinones (BiQ) were originally synthesized as a new class of HIV integrase inhibitors but have shown integrase-independent cytotoxicity in acute lymphoblastic leukemia cell lines suggesting their use as potential anti-neoplastic agents. The mechanism of this cytotoxicity is unknown. In order to gain insight into the mode of action of binaphthoquinones we performed a systematic high-throughput screen in a yeast isogenic deletion mutant array for enhanced or suppressed growth in the presence of binaphthoquinones.

**Methodology/Principal findings:**

Exposure of wild type yeast strains to various BiQs demonstrated inhibition of yeast growth with IC_50_s in the µM range. Drug sensitivity and resistance screens were performed by exposing arrays of a haploid yeast deletion mutant library to BiQs at concentrations near their IC_50_. Sensitivity screens identified yeast with deletions affecting mitochondrial function and cellular respiration as having increased sensitivity to BiQs. Corresponding to this, wild type yeast grown in the absence of a fermentable carbon source were particularly sensitive to BiQs, and treatment with BiQs was shown to disrupt the mitochondrial membrane potential and lead to the generation of reactive oxygen species (ROS). Furthermore, baseline ROS production in BiQ sensitive mutant strains was increased compared to wild type and could be further augmented by the presence of BiQ. Screens for resistance to BiQ action identified the mitochondrial external NAD(P)H dehydrogenase, *NDE1*, as critical to BiQ toxicity and over-expression of this gene resulted in increased ROS production and increased sensitivity of wild type yeast to BiQ.

**Conclusions/Significance:**

In yeast, binaphthoquinone cytotoxicity is likely mediated through NAD(P)H:quonine oxidoreductases leading to ROS production and dysfunctional mitochondria. Further studies are required to validate this mechanism in mammalian cells.

## Introduction

Multimeric naphthoquinones are unique molecules, which possess a diverse array of biologic activities including antineoplastic, antiprotozoal and antiviral effects [1]. Their structures are based on two or more naphthoquinone units linked together in different positions. In nature, their synthesis likely involves oxidative coupling of a common naphthol intermediate in the process of oligomerization [2].

One member of this class, conocurvone, was first isolated from the Western Australian smoke bush and has been shown to inhibit the cytopathogenic effects of HIV-1 in human T lymphoblasts [3]. In an effort to synthesize anti-retrovirals, Emadi et al. previously reported the regiocontrolled synthesis of symmetrical and asymmetrical dimeric and trimeric naphthoquinones by using a novel method [4], [5]. Several of the dimeric naphthoquinones (binaphthoquinones) inhibited HIV-1 integrase with ID_50_ (concentration of drug required to inhibit HIV-1 mediated cytopathogenicity in infected cells by 50%) ranging from 1 to 3.5 micromolar [6]. In addition, potent activity of binapthoquinones against non-HIV infected CEM-T_4_ lymphoblastic leukemia cells was observed with TD_50_ (reduction of non-infected cell growth by 50%) ranging from 5 to 8 micromolar, suggesting the presence of other cytotoxic mechanisms for these compounds [6].

The mechanism of binaphthoquinones cytotoxicity is currently unknown however it may be in part related to the presence of quinone moieties. Previously, quinone cytotoxicity has been attributed to a wide array of cellular effects including DNA and protein modification [1], [7], [8], topoisomerase inhibition [9], [10], caspase activation [11], oxidative stress [1], [12] and endoplasmic reticulum stress [13]. Elucidation of the cellular components necessary to protect cells or sensitize them to binaphthoquinones might allow for the enhanced use of these drugs as antiretroviral, antiparasitic or antineoplastic agents. To this end, we carried out genome wide screens for binaphthoquinone sensitivity and resistance in yeast. Yeast *Saccharamyces cerevisiae* share conserved sequences with known and predicted human proteins and provide a powerful model organism in which high throughput genetic screens can be performed [14], [15], [16], [17]. We utilized several different binaphthoquinone analogues and developed a genetic model of their cytotoxicity by performing a systematic high-throughput screen of a yeast isogenic deletion mutant array for drug enhanced or suppressed growth. We then confirmed the validity of the suggested targets genetically.

## Results

### Binaphthoquinones inhibit yeast growth

**Figure 1:**
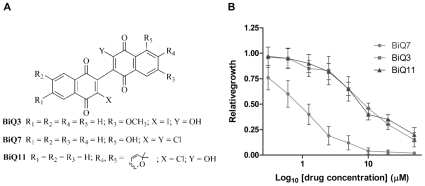
Binaphthoquinones suppress growth of *Saccharomyces cerevisiae*. (A) Chemical structures of dimeric naphthoquinones. (*B*) Determination of the IC_50_ of binaphthoquinones in yeast. Log-phase cultures at an OD_600_ of 0.1 were treated with different concentrations of binaphthoquinones or DMSO over 24 h. Relative growth of wild-type (BY4741) strain in the presence of three of the binaphthoquinones was measured against that of the yeast grown in the presence of DMSO. Growth curves were performed in triplicate and represent the average of three experiments.

To establish whether yeast could be used as a model system for binaphthoquinone cytotoxicity, we first aimed to determine the ability of binaphthoquinones to suppress yeast growth. We selected three different binaphthoquinones to reflect their diverse chemical properties (Fig. 1A). Binaphthoquinone 7 (BiQ7) possesses two chlorine (Cl) atoms on the quinone cores and a hydroxyl group at position 5 on one of the aromatic rings [18]. Binaphthoquinone 3 (BiQ3) is an iodo-hydroxy-binaphthoquinone with a remote methoxyl group on the aromatic ring [6]. Binaphthoquinone 11 (BiQ11) is a pyranylated chloro-hydroxy-binaphthoquinone [6]. All tested binaphthoquinones inhibited growth of wild type yeast (strain BY4741) in liquid cultures in a concentration-dependent manner (Fig. 1B). As the purpose of our yeast screen was to identify drug sensitive strains, we determined the concentrations at which approximately 50% of yeast growth is suppressed (IC_50_) in the wild type strain. Our analysis showed that wild type yeast is particularly sensitive to BiQ7 with an IC_50_ of 0.9±0.2 µM while other tested binaphthoquinones showed IC_50_ in the 7±2 µM range (Fig. 1B).

### Genome-wide growth suppression screening reveals important roles for mitochondria and cellular respiration in the action of binaphthoquinones

For an initial high-throughput phenotypic screen, a set of approximately 5000 commercially available *S. cerevisiae* non-essential isogenic deletion strains were arrayed onto 96 well plates containing either DMSO (no drug control) or a sub-lethal binaphthoquinone concentration at its IC_50_ [15]. Plates were incubated at 23°C and strain growth on DMSO- and binaphthoquinone-treated plates were compared over a period of 24h. The screen was carried out in a duplicate with two different binaphthoquinones (BiQ7 and BiQ3). As expected, wild type yeast in these assays demonstrated a relative growth of 0.6+0.1. Median mutant relative growth was slightly more right-shifted, likely representing conditions in the assay to favor robust growth. Accordingly, a cut off for decreased growth of at least 3 standard deviations below the mean in both screens was chosen to identify binaphthoquinone sensitive strains for further analysis (Supplemental Fig. S1, Supplementary Table S1). As a control we also tested a drug previously analyzed in similar assays, 6-azauracil (6-AU), and obtained a set of sensitive strains similar to those previously reported (data not shown) [19]. While the lists of mutants hypersensitive to BiQ7 and BiQ3 contained many overlapping genes (76 out of 128 genes) (Supplementary Table S1), 6-AU sensitive strains had very little overlap with either binaphthoquinone group (14 out of 128 genes) (Supplemental Fig. S1). The most drug sensitive haploid strains to both binaphthoquinones are listed in Table 1.

**Table 1:** The most sensitive yeast strains to BiQ7 and BiQ3.

BiQ7*	BiQ3*	ORF	Name	Biological Process
0.002	0.002	YML129C	*COX14*	Mitochondrial membrane protein, involved in translational regulation of Cox1p and assembly of cytochrome c oxidase (complex IV)
0.065	0.038	YPL029W	*SUV3*	ATP-dependent RNA helicase, component of the mitochondrial degradosome along with the RNase Dss1p
0.093	0.083	YOR285W	-	Protein of unknown function, localized to the mitochondrial outer membrane
0.074	0.039	YOR194C	*TOA1*	TFIIA large subunit; involved in transcriptional activation, acts as antirepressor or as coactivator
0.020	0.029	YJL166W	*QCR8*	Subunit 8 of ubiquinol cytochrome-c reductase complex, which is a component of the mitochondrial inner membrane electron transport chain
0.064	0.075	YDR487C	*RIB3*	3,4-dihydroxy-2-butanone-4-phosphate synthase (DHBP synthase), required for riboflavin biosynthesis from ribulose-5-phosphate, also has an unrelated function in mitochondrial respiration
0.032	0.063	YMR125W	*GCR3*	Large subunit of the nuclear mRNA cap-binding protein complex; also involved in nuclear mRNA degradation and telomere maintenance
0.042	0.074	YBR003W	*COQ1*	Hexaprenyl pyrophosphate synthetase, catalyzes the first step in ubiquinone (coenzyme Q) biosynthesis
0.005	0.054	YML110C	*COQ5*	2-hexaprenyl-6-methoxy-1,4-benzoquinone methyltransferase, involved in ubiquinone (Coenzyme Q) biosynthesis; localizes to the matrix face of the mitochondrial inner membrane in a large complex with other ubiquinone biosynthetic enzymes
0.036	0.047	YGR255C	*COQ6*	Putative flavin-dependent monooxygenase, involved in ubiquinone (Coenzyme Q) biosynthesis; localizes to the matrix face of the mitochondrial inner membrane in a large complex with other ubiquinone biosynthetic enzymes
0.055	0.073	YER164W	*CHD1*	Nucleosome remodeling factor that functions in regulation of transcription elongation; contains a chromo domain, a helicase domain and a DNA-binding domain; component of both the SAGA and SLIK complexes

*Average relative growth.

**Figure 2:**
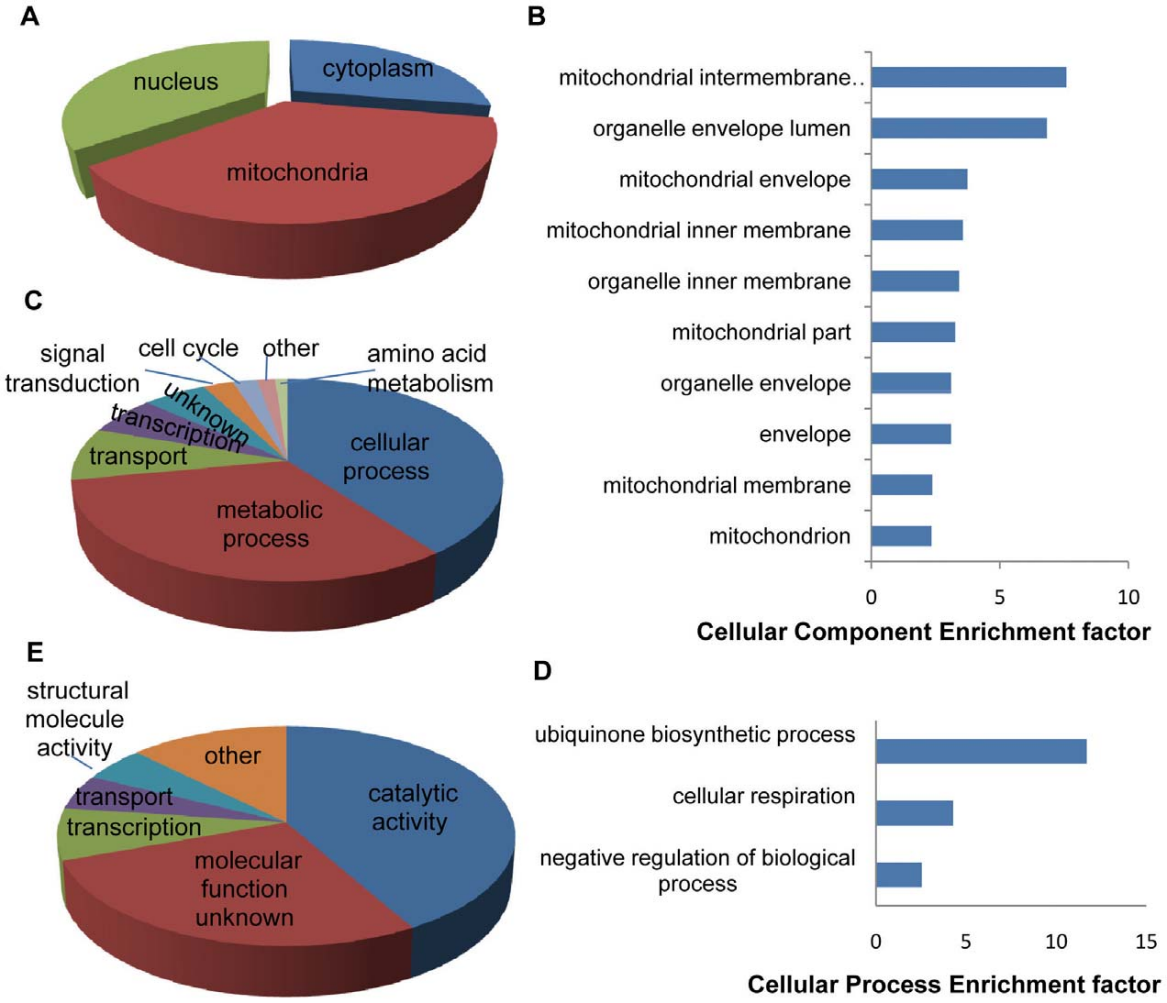
Enhanced sensitivity of mitochondrial and cellular respiration mutants to binaphthoquinones. (*A*) Distribution of all GO Component categories among strains hypersensitive to binaphthoquinones. (*B*) Enrichment factor comparison of significantly enriched cellular component GO categories of deletion strains hypersensitive to binaphthoquinones showing a significant enrichment of mitochondrial mutants. (*C*) Distribution of GO Cellular Process categories among strains hypersensitive to binaphthoquinones. (*D*) Enrichment factor comparison of significantly enriched biological processes of deletion strains hypersensitive to binaphthoquinones demonstrating a significant enrichment of cellular respiration mutants. (*E*) Distribution of GO Function categories among strains hypersensitive to binaphthoquinones.

The strains hypersensitive to binaphthoquinones in both screens were further analyzed for distribution of all (Figures 2A, 2C and 2E) gene ontology (GO) categories among shared mutants [20]. To determine significantly enriched GO categories among hypersensitive mutants, an analysis by GO::TermFinder was carried out. This analysis strongly suggested that strains with defects in mitochondria (p<0.0001), cellular respiration (p<0.01) and ubiquinone biosynthetic process (p<0.05) are particularly sensitive to binaphthoquinones. To rank these categories, we further determined the enrichment factors (fold increase over random enrichment) of the significant GO categories uncovered by GO::TermFinder (Fig. 2B and 2D) [21]. Again mitochondrial mutants and cellular respiration mutants appear significantly enriched (Fig. 2B and 2D). Interestingly, no functional category was significantly enriched with the closest group being oxidoreductases (enrichment factor of 2.34) (p = 0.069).

**Figure 3:**
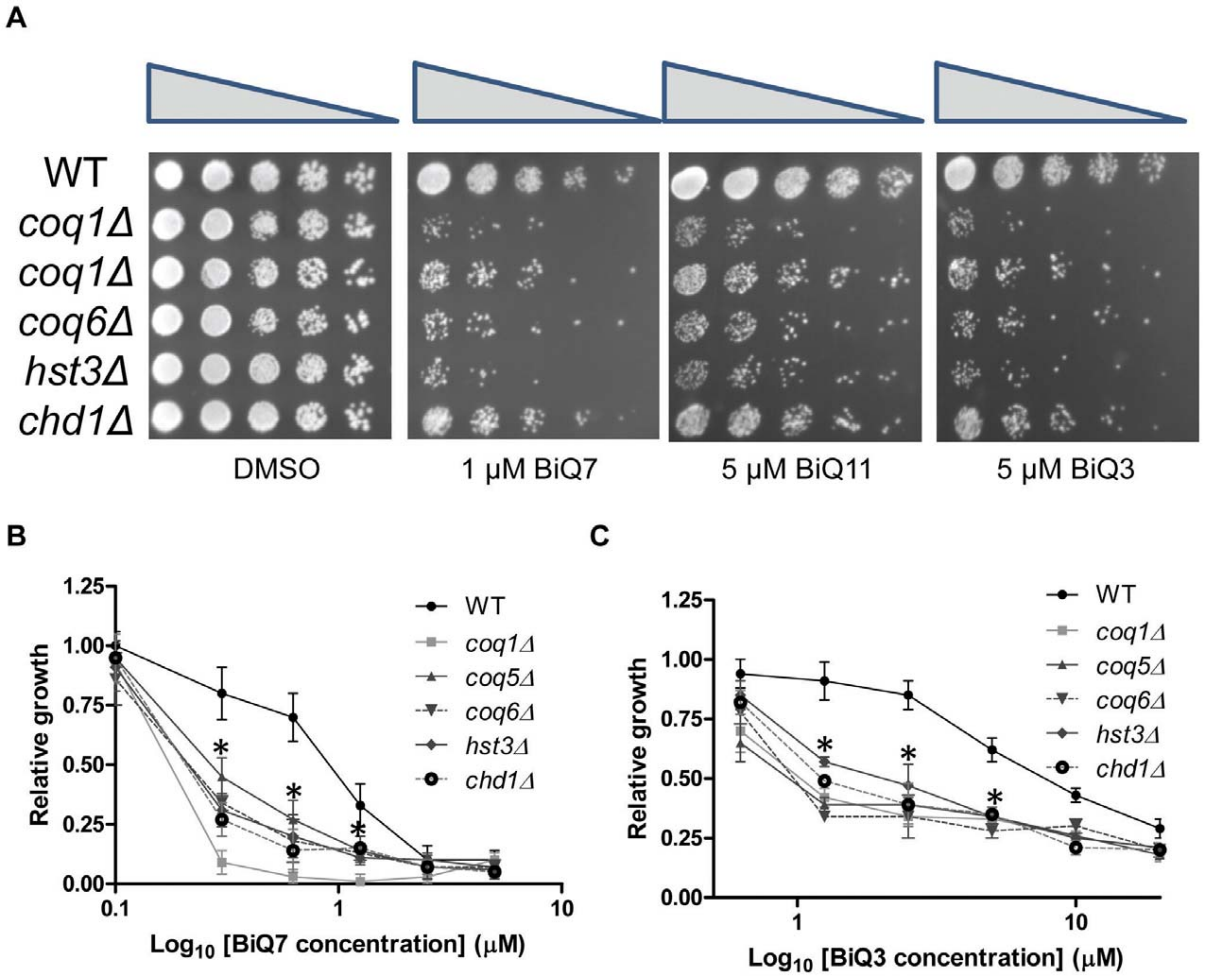
Validation of binaphthoquinone sensitivity of deletion strains identified in the high throughput screen. (*A*) Spot assays of five binapthoquinone sensitive deletion strains. Deletion strains were spotted in five fold serial dilutions on SC-ura plates containing DMSO or BiQ7, BiQ11 or BiQ3 at their IC_50_ concentrations. Spot assays were performed in duplicate in two separate experiments with representative plates shown. (*B,C*) BiQ7 *(B)* or BiQ3 *(C)* sensitivity of several deletion strains identified in the high-throughput screen. Log-phase cultures at an OD_600_ of 0.1 were treated with different concentrations of binaphthoquinones or DMSO over 24h. Relative growth of wild-type and deletion strains in the presence of BiQ7 *(B)* or BiQ3 *(C)* was measured against that of the yeast grown in the presence of DMSO. Growth curves were performed in triplicate and represent the average of three experiments. * = p<0.05 for a difference in the mean compared to wild type (growth compared to wild type was significantly impaired in all deletion mutants tested at 0.3, 0.6 and 1.2 µM of BiQ7 and 1.25, 2.5 and 5 µM of BiQ3).

To confirm the sensitivity of the enriched strains, we individually measured the sensitivity of selected strains to binaphthoquinones by spot assays and relative growth (Fig. 3). Spot assays of tested deletion strains demonstrated increased sensitivity of these strains to BiQs compared to wild-type yeast (Fig. 3A). Furthermore, all tested deletion strains were more sensitive to BiQs than the wild-type control over a range of different concentrations, and this selectivity was lost at higher concentrations of the drug (Fig. 3B and 3C).

### Binaphthoquinones inhibit yeast growth in nonfermentable media and depolarize the mitochondrial membrane

**Figure 4:**
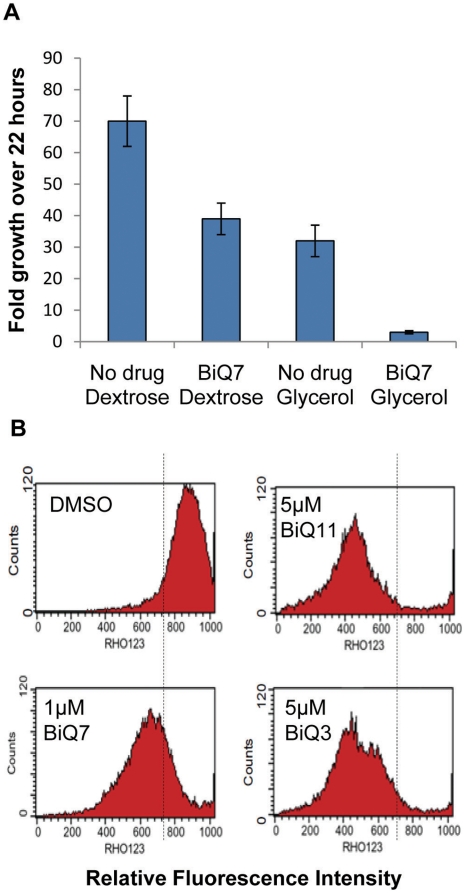
Binaphthoquinones inhibit yeast respiratory growth by depolarizing the mitochondrial membrane. (*A*) BiQ7 inhibits yeast growth in nonfermentable media. Yeast growth is severely repressed in glycerol-containing media in the presence of 1 µM BiQ7 (p<0.001 for differences between all experimental categories except dextrose with drug and glycerol alone). (*B*) Binaphthoquinones depolarize mitochondrial membrane. The wild type yeast was treated at IC_50_ concentrations of binaphthoquinones (1, 5 and 5µM of BiQ7, BiQ11 and BiQ3 respectively) for 2h and then incubated with rhodamine 123. The peak shift toward the left represents a decrease of fluorescence signal of rhodamine 123 in the yeast, indicating the loss of mitochondrial membrane potential in the presence of BiQs as compared to no drug (DMSO) control.

Yeast are able to grow either anaerobically or aerobically by utilizing fermentable or nonfermentable carbon sources, respectively. In the presence of a fermentable carbon source, such as glucose (dextrose), yeast preferentially adopt glycolysis to generate energy even under aerobic conditions and can grow normally even when mitochondrial respiration level is minimal [22]. In order to further determine whether binaphthoquinone toxicity occurs via interference with mitochondrial function, we compared yeast growth in the presence or absence of BiQ7 in a dextrose containing media or in media where glucose was replaced with glycerol, a nonfermentable carbon source. Yeast growth in glycerol-containing media was severely inhibited by BiQ7 indicating that binaphthoquinones affect mitochondrial function (Fig. 4A).

Enhanced sensitivity of yeast mitochondrial mutants to binaphthoquinones as well as the observation of severe growth inhibition in nonfermentable media in the presence of BiQ7 encouraged us to examine whether the yeast mitochondrial membrane potential (critical to mitochondrial functions such as oxidative phosphorylation, lipid and pyrimidine synthesis) is affected by binaphthoquinones. Treatment of yeast with binaphthoquinones led to extensive loss of membrane potential, as evidenced by the decreased rhodamine 123 uptake into the mitochondria (Fig. 4B). This effect was observed with yeast grown in either glycerol or dextrose-containing media, but the degree of membrane potential loss was greater in the glycerol-containing media (data not shown).

### Binaphthoquinones generate reactive oxygen species

Monoquinones are believed to generate superoxide radicals and oxidative stress by undergoing a futile redox cycle in which carbonyl groups are reduced by reductases to a semiquinone (Q^.−^) radical and subsequently to a hydroquinone (QH_2_), which then rapidly undergoes a two-step oxidation back to the parent compound. This redox cycling and oxygen activation, in theory, results in cytotoxic levels of ROS [1].

**Figure 5:**
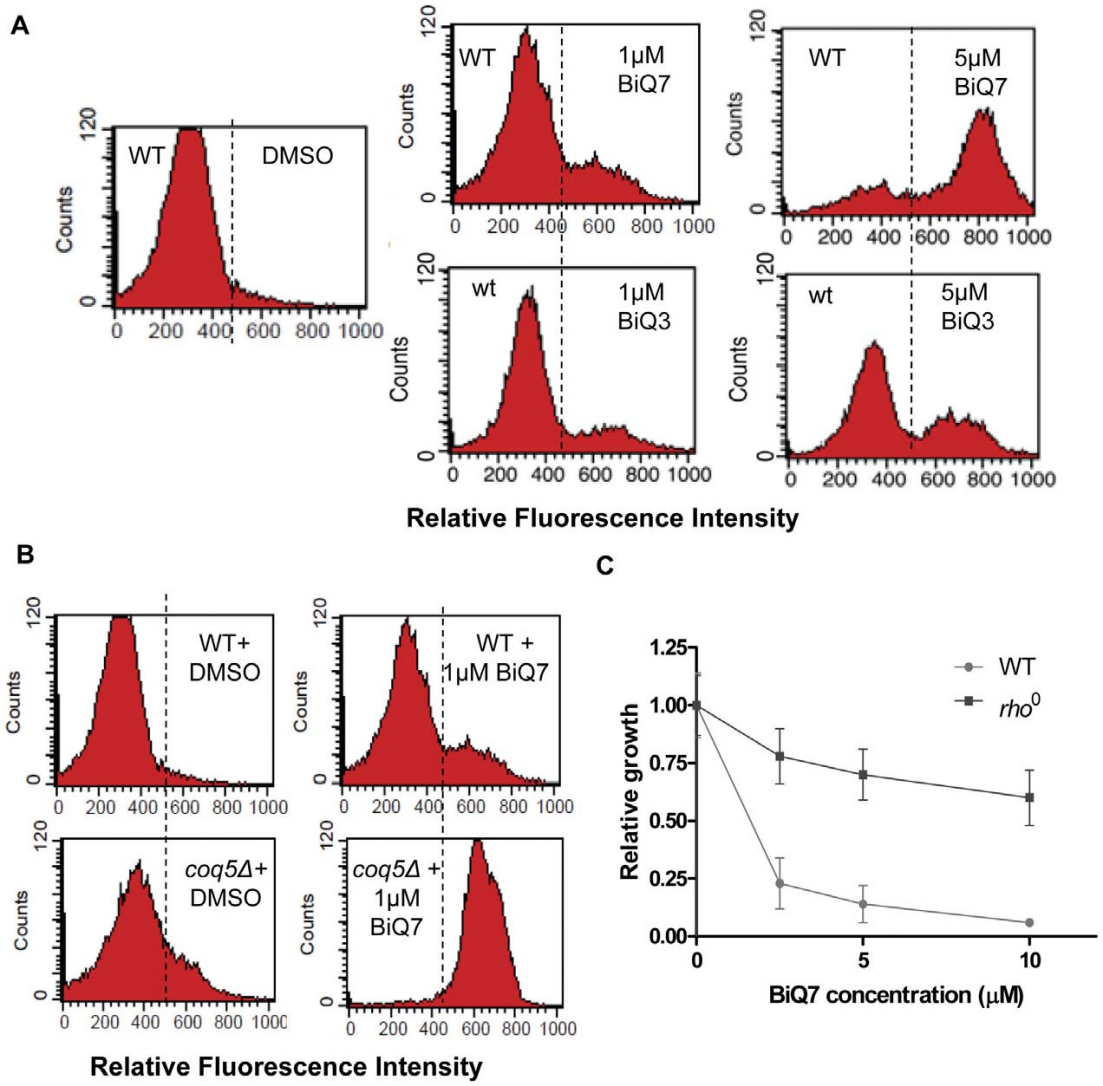
Binaphthoquinones generate reactive oxygen species in yeast. (*A*) Binaphthoquinones generate ROS in a concentration dependent manner. Yeast wild type strain was treated with DMSO, BiQ7 or BiQ3 at increasing concentrations for 2h and incubated with DHR 123. Experiments were performed three times with similar results. (*B*). Enhanced generation of ROS in the BiQ hypersensitive mutant *coq5*Δ in the presence and absence of binaphthoquinone. Yeast wild type and *coq5*Δ cells were treated with 1 µM BiQ7 or DMSO for 2h and incubated with DHR 123. The experiment was performed three times with similar results. (*C*) Mitochondrial deficient yeast, *rho^0^*, are resistant to binapthoquinones. Log-phase cultures of wild-type or *rho^0^* yeast at an OD_600_ of 0.1 were treated with DMSO or different concentrations of BiQ7 for 24 (wild type) and 48 (*rho^0^*) h. Growth curves were performed in triplicate and represent the average of three experiments. P<0.05 for differences between wild-type and *rho^0^* yeast at all concentrations of drug.

To further assess production of ROS, wild type yeast were exposed to 0, 1, or 5 µM of binaphthoquinones followed by incubation with dihydrorhodamine 123 (DHR 123) for 1 h. Oxidation of DHR 123 to rhodamine 123 produces a fluorescent signal that can be measured. Treatment of yeast with BiQ7 or BiQ3 resulted in the oxidation of DHR 123 in a concentration-dependent manner (Fig. 5A). We additionally tested one of the hypersensitive strains, *coq5Δ* (a para-benzoquinone methyltransferase, involved in ubiquinone biosynthesis and localized on the matrix face of the mitochondrial inner membrane), for ROS production. ROS production was increased in this mutant strain compared to wild type both in the presence and absence of BiQ7 (Fig. 5B). Previously, several groups had demonstrated that cells lacking mitochondrial DNA (*rho* ^0^) were incapable of generating reactive oxygen species via redox mechanisms [23], [24]. We hypothesized that if BiQ toxicity was mediated through the generation of ROS, *rho* ^0^ strains would demonstrate resistance. Accordingly, incubation of *rho* ^0^ yeast with BiQ7 over a range of concentrations showed greatly abrogated effects of the drug in this strain (Fig. 5C).

We additionally tested whether the addition of the antioxidant, N-acetyl cysteine (NAC) could neutralize the effects of BiQs. Co-incubation of yeast with NAC resulted in decreased ROS formation and increased relative growth of multiple BiQ sensitive strains (Supplemental Fig. S2). While NAC may serve to diminish binaphthoquinone cytotoxicity by neutralizing ROS through increased glutathione production, it also could exert its actions by interacting directly with binaphthoquinones or their derivatives to reduce their cytotoxicity [13], [25]. To test for whether direct interaction between NAC and BiQs occurs, we performed mass spectrometry on BiQ7 (MW 398 Da) and NAC (MW 163 Da) both separately and after co-incubation (predicted reacted products at 526 Da or 561 Da). A product at 526 Da was present in the coincubated sample which could be fragmented into components of 361 and 162 Da, suggesting that NAC can interact directly with binaphthoquinones (data not shown).

### NAD(P)H:quinone oxidoreductase type II, *NDE1*, plays an important role in the action of binaphthoquinones

**Figure 6:**
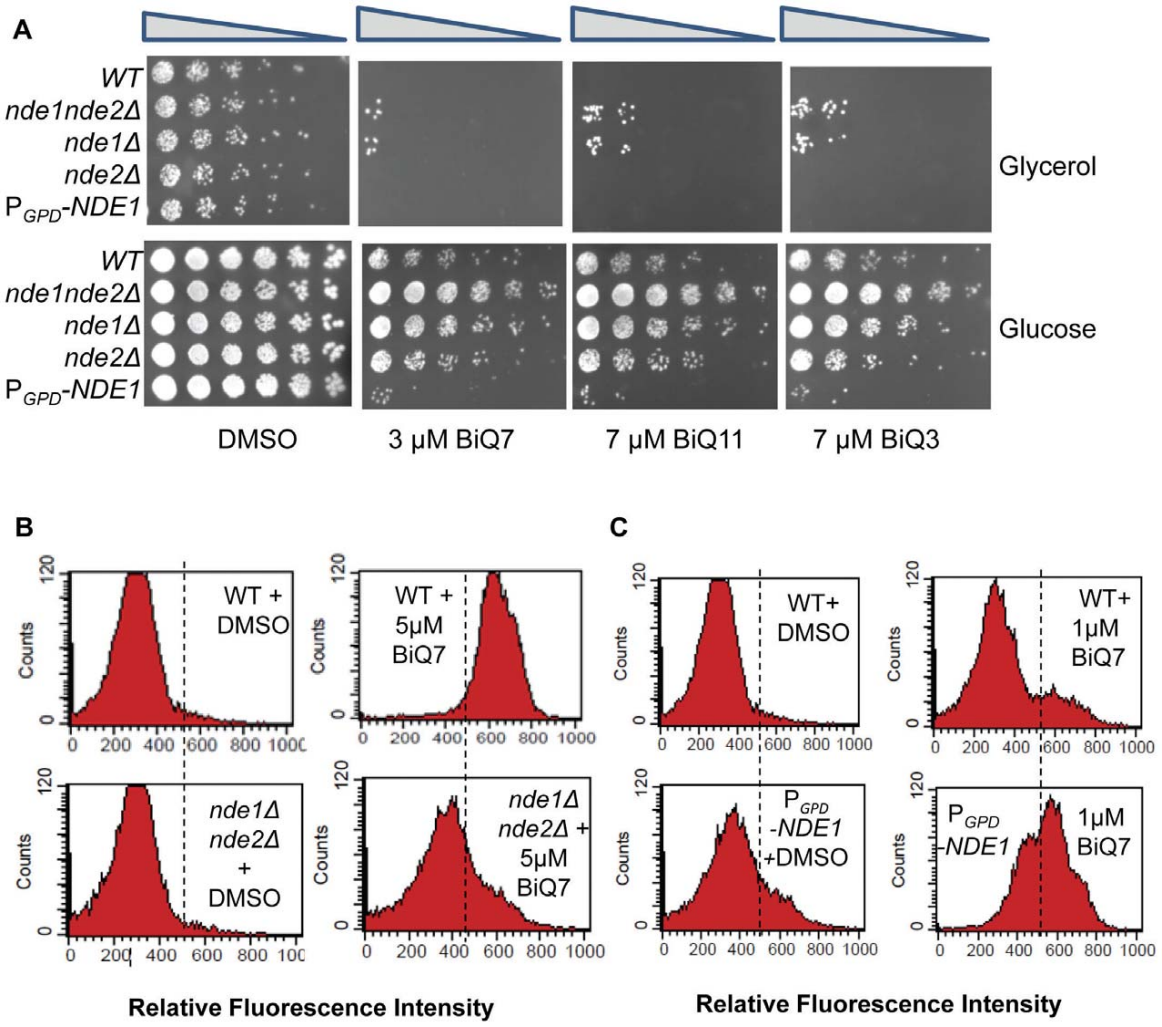
*NDE1* plays a critical role in the action of binaphthoquinones. (*A*) Dependence of yeast sensitivity to BiQs on *NDE1* in glucose and glycerol containing media. Spot assays of wild-type, *nde1nde2*Δ, *nde1*Δ, *nde2*Δ and wild type yeast overexpressing *NDE1* driven by a GPD promoter (P*_GPD_*-*NDE1*) were spotted in five fold serial dilutions on plates containing glycerol or glucose and either DMSO or BiQ7, BiQ11 or BiQ3 at their IC_50_ concentrations. Spot assays were performed in duplicate in two separate experiments with representative plates shown. (*B*) Decreased generation of ROS in the *nde1*Δ*nde2*Δ mutant following exposure to 5 µM BiQ7. (*C*) Increased generation of ROS in a yeast overexpressing *NDE1* (P*_GPD_*-*NDE1*) following exposure to 1 µM BiQ7. Yeast strains were treated with binaphthoquinones or DMSO for 2h and incubated with DHR 123. The experiment was performed three times with similar results.

Our initial screens to define BiQ sensitive mutants were designed to allow for robust yeast growth and thus be more likely to identify drug sensitive strains. While this method was effective for defining mutants with decreased survival, it proved limited in the identification of BiQ resistant mutants. For example, while the same drug sensitive strains were reproducibly found in repeat experiments of this screen (Fig 2), strains suggested to be resistant by this assay (3 SD above the mean) were not reproducibly consistent. This may be due to the high ODs registered by BiQ resistant mutants which are out of the linear range for absorbance. Accordingly, to screen for yeast mutants resistant to the binaphthoquinones we exposed the yeast deletion library grown in glycerol-containing agar plates to 3 µM of BiQ7, or 7 µM of BiQ11, 7 µM of BiQ3 or DMSO (Supplemental Table S2). Glycerol plates were used as wild-type yeast growth on glycerol plates is severely inhibited by exposure to BiQs thus reducing background. Only the deletion mutant *nde1Δ*, lacking a mitochondrial NADH dehydrogenase, showed resistance to all tested binaphthoquinone molecules (Supplemental Table S2, Fig. 6A and Supplemental Fig. S3). As *NDE1* contains sequence similarities to *NDE2* (another mitochondrial NADH dehydrogenase) [26], [27], we proceeded with individual testing of mutants lacking these genes either separately or in combination over a wide concentration range of all three binaphthoquinones (Supplemental Fig. S3) [28]. The *nde1Δ* and *nde1Δ nde2Δ* strains showed enhanced resistance to BiQs (Figs. 6A and Supplemental Fig. S3). In contrast, the *nde2Δ* mutant showed no significant resistance to the binaphthoquinones (Figs. 6A and S3). This may be explained by the fact that at the cytosolic side of the inner mitochondrial membrane Nde1p is the major NADH-dehydrogenase [26]. Interestingly, deletion of main inner membrane NADH dehydrogenase *NDI1* showed increased sensitivity to binaphthoquinones (Supplemental Fig. S3). We therefore concluded that the presence of some NADH dehydrogenases might enhance binaphthoquinone toxicity. Indeed, overexpression of *NDE1* under a *GPD* (glyceraldehyde 3-phosphate dehydrogenase) promoter [29] in the parental BY4741 background (wild type) resulted in increased sensitivity to binaphthoquinones (Fig. 6A, Supplemental Fig. S3D–F). Similarly the *nde1*Δ *nde2*Δ deletion strain had diminished ROS production as compared to wild type strain in cells treated with 5 µM of BiQ7 (Fig. 6B), while overexpression of *NDE1* augmented ROS production in the wild type strain as compared to vector alone control when treated with 1 µM of BiQ7 (Fig. 6C).

**Figure 7:**
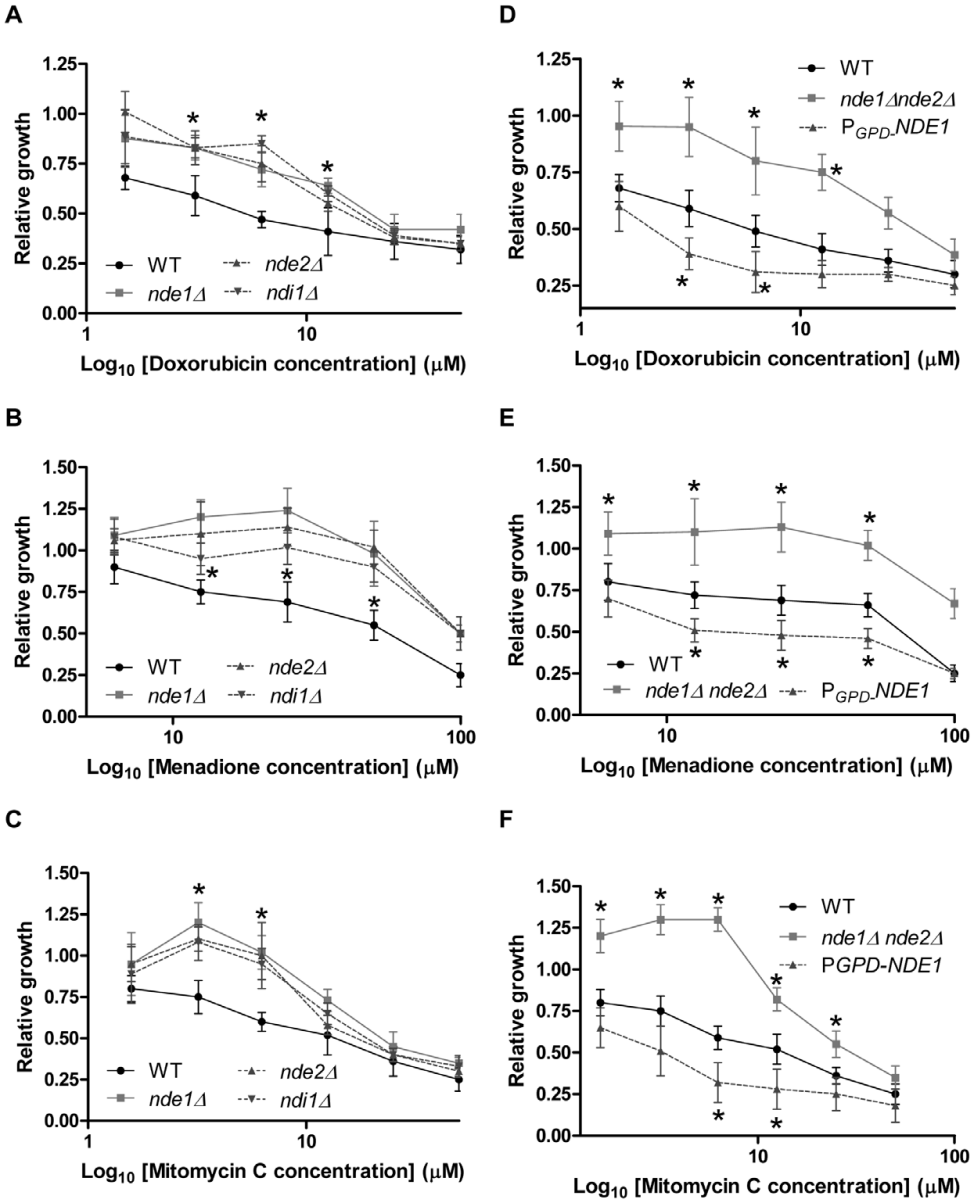
The effects of several quinone based drugs on the relative survival of *NDE1* mutants. (*A,D*) doxorubicin, (*B,E*) mitomycin C, (*C,F*) menadione. Log-phase cultures at an OD_600_ of 0.1 were treated with different concentrations of BiQ7 or DMSO over 17 h. Relative growth of wild-type and mutant strains in the presence of binaphthoquinones was measured against that of the yeast grown in the presence of DMSO. Growth curves were performed in triplicate and represent the average of three experiments. *P<0.05 for differences between wild-type and *NDE1* over-expressing or mutant yeast.

We next tested whether the cytotoxicity of several well known quinone-containing drugs including doxorubicin, mitomycin C and menadione, was partially dependent on *NDE1*. These drugs showed sensitivity and resistance patterns similar to that of binaphthoquinones (Fig. 7). In contrast to binaphthoquinones, however, the *ndi1*Δ strain was resistant to these drugs (Fig. 7A–C).

## Discussion

In this report, we utilized a yeast model to decipher the cytotoxic action of binaphthoquinones. We showed that binaphthoquinones are capable of depolarizing the mitochondrial membrane and that interference with cellular respiration increases the sensitivity of the cells to binaphthoquinones. Furthermore, we demonstrated that binaphthoquinones generate ROS and that the toxic effects of many binaphthoquinones is dependent on NAD(P)H:quinone oxidoreductase type II, *NDE1*.

Our screen results underscore the importance of intact cellular respiration in diminishing the negative effects of binaphthoquinones on cellular growth. Among the most sensitive strains, several are involved in ubiquinone synthesis. Ubiquinone is a monobenzoquinone present in the mitochondria of most eukaryotic cells as a part of the electron transport chain which participates in aerobic cellular respiration [30]. Ubiquinone possesses antioxidant properties and disruption of ubiquinone synthesis leads to increased ROS production as seen in our binaphthoquinones sensitive mutants [31]. Disruption of electron flow, such as would occur in ubiquinone deficient cells, would be expected to result in a backlog of electrons along the NADH utilizing electron transport pathway and this likely synergizes with increased ROS production by biquinones to cause cytotoxicity. Accordingly we observed substantial increases in ROS production compared to wild type when the ubiquinone synthesis mutant *coq5*Δ was treated with binaphthoquinones.

To determine whether binaphthoquinones affect the mitochondrial membrane potential, we studied the ability of BiQs to depolarize mitochondrial membrane potential in both dextrose and glycerol-containing media. All tested BiQs were capable of depolarizing mitochondrial membrane in both media, though the effect was stronger in nonfermentable media. Attenuation of the effect of BiQs on depolarizing mitochondrial membrane potential by dextrose is possibly due to the partial repression of the activity of respiration enzymes, including *NDE1*, by glucose [28]. Interestingly, at IC_50_ concentrations of binaphthoquinones the inhibition of respiratory chain is most noticeable in BiQ11. This might be due to the presence of the pyran ring in this molecule which increases its lipophilicity.

Furthermore, binaphthoquinones were found to be capable of inducing oxidative stress in yeast cells by increasing ROS production. Additionally, binaphthoquinone cytotoxicity was abolished in mitochondrial DNA deficient yeast strains which lack critical respiratory chain catalytic subunits and have diminished ROS production [24].

A screen for deletion mutants resistant to binaphthoquinones identified *NDE1*, a mitochondrial external NADH dehydrogenase (a type II NAD(P)H:quinone oxidoreductase) that catalyzes the oxidation of cytosolic NADH. Nde1p and Nde2p function primarily to provide cytosolic NADH to the mitochondrial respiratory chain [26], [27]. Our findings suggest *NDE1* is involved in bioactivation of BiQs. Deletion of *NDE1* increased resistance of yeast to binaphthoquinone action, while overexpression of *NDE1* has an opposite effect. Interestingly, and in contrast to *nde1*Δ, the *ndi1*Δ strain demonstrated sensitivity to BiQs and this was not reproduced in relationship to other quinone containing drugs (doxorubicin, mitomycin C and menadione). It is possible that binaphthoquinones, due to their larger size and varied hydrophilicity are less accessible to the internal side of the mitochondrion. In addition, *NDE1* may play a unique role in the bioactivation of BiQs. Indeed, another oxidoreductase, *LOT6*, has been previously described as having the ability to detoxify quinones in yeast but in our studies, growth of the *lot6*Δ mutant did not differ significantly from the wild type control when exposed to a range of binaphthoquinone concentrations (data not shown) [32].

**Figure 8:**
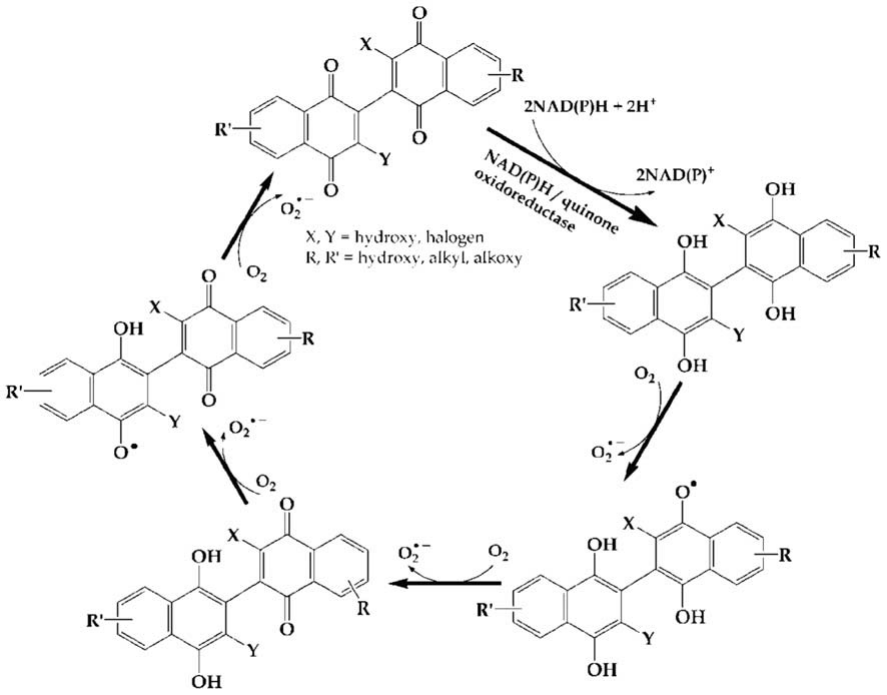
Proposed mechanism of binaphthoquinones redox cycling. Our data suggest that binaphthoquinones undergo oxidoreductase-dependent redox cycling which results in ROS generation and cytotoxic action of these molecules. Binaphthoquinones are reduced to the hydroquinone forms, which are unstable and through four one-electron oxidation steps converts back to the parent molecule. Molecular oxygens act as electron acceptors and convert to superoxide radicals.

In contrast to the mitochondria of fungi and plants, mammalian mitochondria do not harbor external NADH dehydrogenases and instead depend on redox shuttle mechanisms to couple the oxidation of cytosolic NADH to internal NADH dehydrogenases [33]. Despite this, the main enzymes involved in quinone metabolism in human cells belong to the NAD(P)H:quinone acceptor oxidoreductase (NQO) gene family and, with respect to binaphthoquinone metabolism, may function similarly to *NDE1*. For example, in last two decades, attention has been given to beta-lapachone, an ortho-naphtoquinone antineoplastic drug, and other quinone based drugs which take advantage of NQO1 overexpression in human cancers and selectively target them [34], [35], [36], [37]. NQO1 reduces beta-lapachone to an unstable hydroquinone that rapidly undergoes a two-step oxidation back to fully oxidized parent molecule, perpetuating a futile redox cycle. Deficiency or inhibition of NQO1 renders cells resistant to beta-lapachone [36]. Additionally, our preliminary data in human cells overexpressing NQO1 show increased sensitivity of these cells to both beta-lapachone and binaphthoquinones (unpublished results). Based on these findings, we propose that fully oxidized binaphthoquinones undergo enzymatic reduction by NAD(P)H:quinone oxidoreductases to fully reduced form of bi-hydronaphthoquinones. Subsequently, through a series of oxidation steps, hydronaphthoquinones convert to semiquinones and ultimately back to oxidized binaphthoquinones. These stepwise oxidations culminates in ROS generation (Fig. 8).

Compared to other binaphthoquinones and quinones tested in this study, BiQ7 shows the most potent cytotoxic activity. Several observations might explain this phenomenon. Because BiQ7 possesses a hydroxyl group at the 5-position, it is able to form an intramolecular hydrogen bond with the carbonyl group's oxygen. Interestingly, previous studies of monomeric naphthoquinones with a hydroxyl group at position 5 on the aromatic ring have also shown an increase in toxicity toward rat hepatocytes compared to other naphthoquinones [38]. There are several other potential factors that might have contributed to the higher potency of BiQ7 and quinone drugs with hydroxyl group at 5-position: 1) increased efficiency of redox cycling in BiQ7, 2) increased stability of the semiquinone derived from 5-hydroxy-1,4-naphthoquinone as compared to 1,4-naphthoquinone, which may lead to a higher semiquinone concentration and thereby a higher rate of autoxidation, 3) stabilization of other BiQs after deprotonation of their core hydroxyl groups, which BiQ7 lacks, and donation of the electrons from the deprotonized oxygen to the quinone ring to form tautomers, and 4) better utilization of oxidoreductases [1], [34].

In conclusion, here we use high throughput screens in yeast to elucidate the basic cellular mechanisms mediating binaphthoquinone cytotoxicity. We find that treatment with binaphthoquinones depolarizes mitochondrial membranes and results in the generation of ROS. Accordingly, binaphthoquinone cytotoxicity can be abrogated in yeast mitochondrial DNA deficient mutants. Furthermore, we demonstrate the dependency of binaphthoquinone cytotoxicity on *NDE1* and the ability to sensitize yeast to binaphthoquinones by overexpression of this enzyme. These mechanisms are likely paralleled in mammalian cells and manipulation of these pathways may allow for enhanced use of these drugs as therapeutic agents.

## Materials and Methods

### Ethics Statement

N/A

### Chemicals

Dimeric naphthoquinones [(3,3′-dichloro-8-hydroxy-2,2′-binaphthalenyl-1,4,1′,4′-tetraone (binaphthoquinone #7), 3-Hydroxy-3′-iodo-7-methoxy-2,2′-binaphthalenyl-1,4,1′,4′-tetraone (binaphthoquinone #3) and 8-(3-Chloro-1,4-dioxo-1,4-dihydronaphthalen-2-yl)-9-hydroxy-3,3-dimethyl-3*H*-benzo[*f*]chromene-7,10-dione (binaphthoquinone #11)] were synthesized and characterized by us as previously described and dissolved in DMSO. N-acetylcysteine (NAC), doxorubicin, menadione, mitomycin C, Rhodamine 123 and 123-dihydrorhodamine (123 DHR) were purchased from Sigma–Aldrich (St. Louis, MO).

### Yeast Strains, Media, and Genetic Manipulations

Haploid deletion mutants were generated by the International Deletion Consortium and were obtained from American Type Culture Collection (ATCC) [15]. Yeast strain BY4741 (*MATa his3Δ1 leu2Δ0 met15Δ0 ura3Δ0*) was used as wild type strain. Yeast were grown on synthetic complete (SC) media supplemented with amino acids and lacking uracil (2% glycerol was substituted for glucose as a carbon source as indicated). An isogenic *rho* ^0^ mutant was generated by ethidium bromide treatment as described previously [39].

### Drug Sensitivity Screen and Individual Growth Rate Analysis

The deletion mutant arrays (DMA) containing a yeast artificial chromosome carrying the URA3 marker were propagated on SC medium lacking uracil. The DMA were transferred by hand with a model MC96 96-pin replicator (Dan-Kar Corp, Woburn, MA) into 96-well plates containing 100 microliters of liquid media and were grown overnight at 23°C to saturation. Saturated cultures compensated for differences in growth rate between strains, to ensure that roughly equal amounts of cells were deposited on the agar plates. After 24h, the DMA were pinned onto 96-well plates and were allowed to grow until the OD_600_ of 0.01 was reached at which point media with either DMSO or BiQs in a final concentration of of IC_50_ were added. Growth rates were measured by determination of OD_600_ as a function of time by using a VersaMax plate reader (Molecular Device, Sunnyvale, CA). Growth curves were carried out in triplicate and curves shown are averages of three experiments with error bars representing one STD. Individual growth rate was carried out in a similar manner with a wider spectrum of drug concentrations. DMSO was used as a no drug control. Where indicated, 100 µg/mL NAC was added. Spot assays were performed by growth of yeast strains to an OD_600_ of 1 and then serially spotting them at 5 fold dilutions onto glucose or glycerol containing plates at indicated concentrations of BiQs.

### Database

For further information, refer to the *Saccharomyces* Genome Database (SGD), which can be accessed at www.yeastgenome.com. For gene ontology analyses, we used the GO annotation for yeast gene products curated by SGD. Specifically, we determined significantly enriched GO annotations among the genes within each subcluster using GO::TermFinder (http://www.yeastgenome.org/cgi-bin/GO/goTermFinder.pl) and GO::SlimMapper (http://www.yeastgenome.org/cgi-bin/GO/goSlimMapper.pl) [40]. Enrichment factor was calculated by using the formula (*a*/*b*)/(*c*/*d*), where *a* is the number of mutants in a particular GO category with decreased growth, *b* is the total number of genes with decreased growth, *c* is the total number of genes in a particular GO category and *d* is the total number of genes across all GO categories. We defined significant enrichment as at least 3 fold above random enrichment (enrichment factor of 1) [21].

### Drug Resistance Screen

DMA were arrayed onto glycerol containing plates with DMSO or BiQ at the indicated concentration at high density by using a 96-pin tool that spots 20 nanoliter or 250 cells. Strains were pinned in duplicate with a density of approximately 864 per plate. Pins were flame sterilized. Plates were grown at 23°C and strains were scored for growth, slow growth, or no growth, compared with no drug over a period of 4 days.

### Gene deletion and ectopic expression

The double mutant *nde1Δ nde2Δ* was generated by deleting the open reading frame of *NDE2* from the *nde1Δ* strain (BY4741) by using homologous recombination [41]. Gene deletion was confirmed by PCR. The amplified open reading frame of *NDE1* gene was cloned into a *GPD*-promoter driven expression vector, p415GPD. Yeast transformation was done by the lithium acetate method. Transformed cells were selected on SC-leucin plates. Sequences for primers used for generation of knock-out strain and expression vector are available upon request. The identity of all strains used for individual experiments were confirmed by uptag and downtag amplification and sequencing as described previously [29].

### Assay of the electrochemical potential

After treatment in 20 mM HEPES buffer (pH 7.4) containing 50 mM glucose, 1 ml of the yeast suspension was incubated with 2 µM Rh123 (rhodamine 123) for 30 minutes at at 23° Celsius, washed, and then resuspended in 100 microliter PBS. Propidium iodide (PI) was then added 10 minutes prior to analysis by flow cytometry. Mitochondrial electrochemical potential is correlated to the fluorescence intensity of Rho123 (with decreased fluorescence signifying loss of the mitochondrial electrochemical potential). Flow cytometry was performed using a FACS Calibur (Becton Dickinson, San Jose, California, United States) with excitation at 488nm and emission read using a 525–550nm filter (FL1). PI stained, dead cells were excluded from analysis.

### Flow cytometric analysis of ROS production

To assess production of radical oxygen species, cells were exposed to BiQs for 2h at 23° Celsius, washed and then incubated with dihydrorhodamine 123 (DHR 123) for 1 h at 23° Celsius and then propidium iodide (PI) for 10 minutes. Cells were analyzed by a FACS Calibur at excitation and emission settings of 488 and 525–550 nm (filter FL1), respectively. PI stained, dead cells, were excluded from analysis.

### Mass Spectrometry

Masses of BiQ7 and NAC either invidually or after coincubation at room temperature for 2 hours was determined by MALDI-TOF and QSTAR mass spectrometry performed by the Johns Hopkins Proteomics Core Facility [42].

## Supporting Information

Figure S1 Relative growth distribution of yeast mutants following exposure to drugs. *(A)* BiQ3, *(B)* BiQ7 and *(C)* 6-AU. Yeast deletion mutant array log-phase cultures at an OD_600_ of 0.1 were treated with DMSO or BiQ3, BiQ7, or 6-azauracil (6-AU) at 5µM, 1µM or 2 mM respectively over 24 h. Relative growth of wild-type (BY4741) strain in the presence of drugs were measured against that of the yeast grown in the presence of DMSO. Growth curves were performed in duplicate. The number of mutants at indicated relative growth values was plotted. Broken lines indicate values 3 SD below the mean. *(D)* Venn's diagram of hypersensitive mutants shared between BiQ3, BiQ7 and 6-AU.
(0.28 MB TIF)

Figure S2 Neutralization of BiQ free radical generation and cytotoxicity by treatment with N-acetylcysteine (NAC). *(A)* Abrogation of BiQ dependent ROS generation by coincubation with NAC. Yeast wild type strain was treated with BiQ7 at increasing concentrations in the presence or absence of NAC for 2h and then incubated with DHR 123 for 1h. Experiments were performed three times with similar results. *(B)* Suppression of yeast growth by binaphthoquinone is neutralized by addition of NAC. Decreased growth of wild type and sensitive yeast mutants was rescued by addition of 100 µg/mL of NAC. Log-phase cultures at an OD_600_ of 0.1 were treated with different concentrations of BiQ7 or DMSO over 24h. Relative growth of wild-type and mutant strains in the presence of BiQ7 was measured against that of the yeast grown in the presence of DMSO. Growth curves were performed in triplicate and represent the average of three experiments. *p<0.05 for differences between wild-type and *NDE1* over-expressing or mutant yeast.
(0.58 MB TIF)

Figure S3 Deletion of *nde1 nde2* enhances resistance to BiQs while overexpression of NDE1 enhances sensitivity to BiQs. *(A,D)* BiQ7 *(B,E)* BiQ3, *(C,F)* BiQ11. Log-phase cultures at an OD600 of 0.1 were treated with different concentrations of corresponding binaphthoquinones or DMSO over 24h. Relative growth of wild-type and mutant strains in the presence of binaphthoquinones was measured against that of the yeast grown in the presence of DMSO. Growth curves were performed in triplicate and represent the average of three experiments. *p<0.05 for differences between wild-type and mutant yeast.
(0.39 MB TIF)

Table S1 Yeast deletion mutants hypersensitive to the IC50 concentrations of BiQ7 and BiQ3. BiQ7 and BiQ3 hypersensitivity in the deletion strains was measured by assessing growth defect as compared to DMSO by measuring OD600 (complete description in Results section of the text). References that describe haploid deletion strains that are sensitive to BiQ7 and BiQ3 are described in the text (see Results section for detailed description). Gene functions and cellular component of corresponding yeast proteins were obtained from SGD.
(0.05 MB XLS)

Table S2 Yeast deletion mutants resistant to binaphthoquinones. Yeast deletion strains resistant to BiQ7, BiQ11 or BiQ3 at 3, 7 and 7 µM concentrations respectively are listed.
(0.02 MB XLS)
