# Supplementary figures and images for: A Chemical Genetic Screen for Modulators of Asymmetrical 2,2′-Dimeric Naphthoquinones Cytotoxicity in Yeast
Source: PLoS One. 2010 May 26;5(5):e10846. doi: 10.1371/journal.pone.0010846 (PMC2877097; doi:10.1371/journal.pone.0010846)

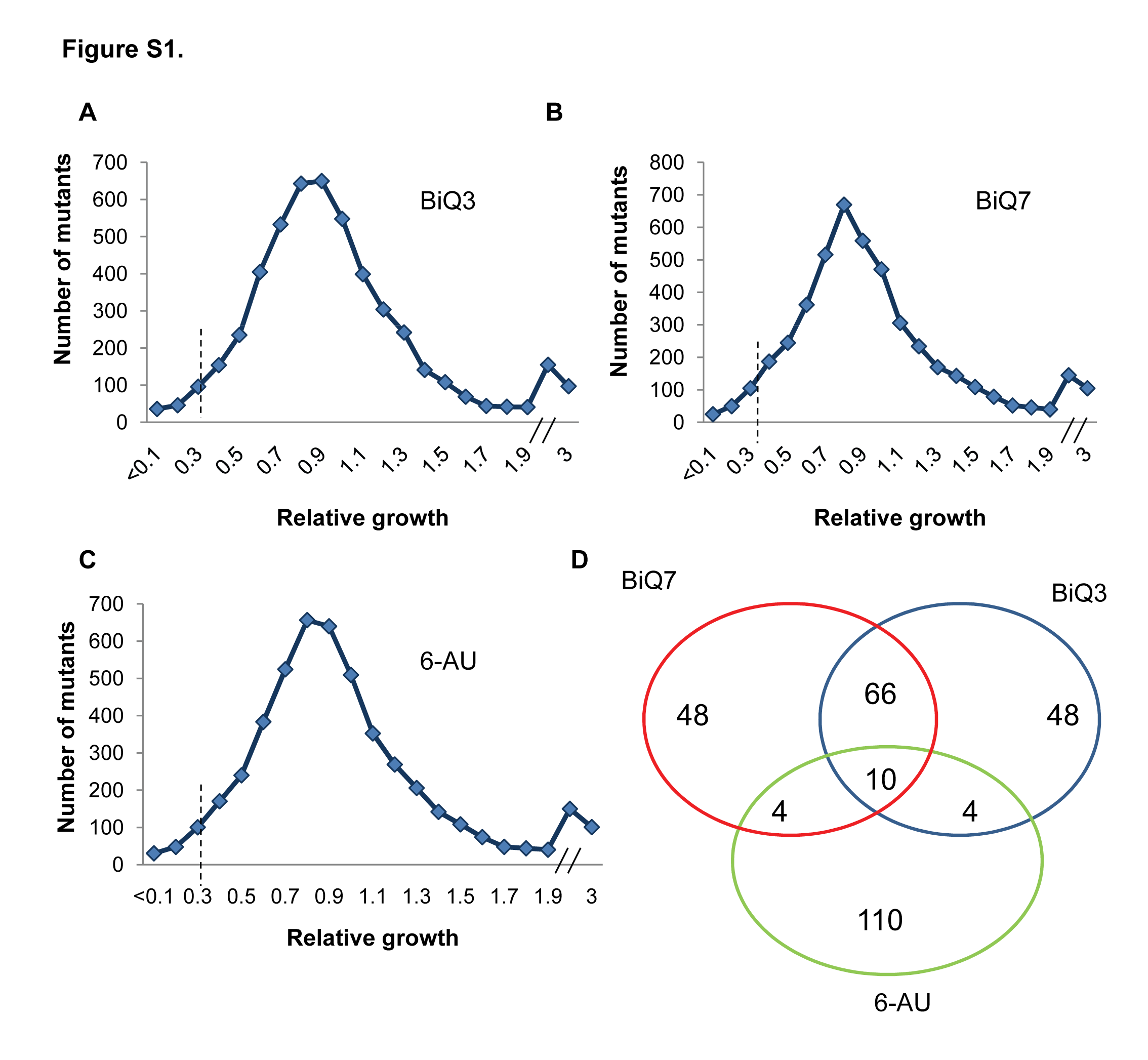

Supplement: Figure S1 — Relative growth distribution of yeast mutants following exposure to drugs. (A) BiQ3, (B) BiQ7 and (C) 6-AU. Yeast deletion mutant array log-phase cultures at an OD600 of 0.1 were treated with DMSO or BiQ3, BiQ7, or 6-azauracil (6-AU) at 5µM, 1µM or 2 mM respectively over 24 h. Relative growth of wild-type (BY4741) strain in the presence of drugs were measured against that of the yeast grown in the presence of DMSO. Growth curves were performed in duplicate. The number of mutants at indicated relative growth values was plotted. Broken lines indicate values 3 SD below the mean. (D) Venn's diagram of hypersensitive mutants shared between BiQ3, BiQ7 and 6-AU. (0.28 MB TIF) [file pone.0010846.s001.tif]

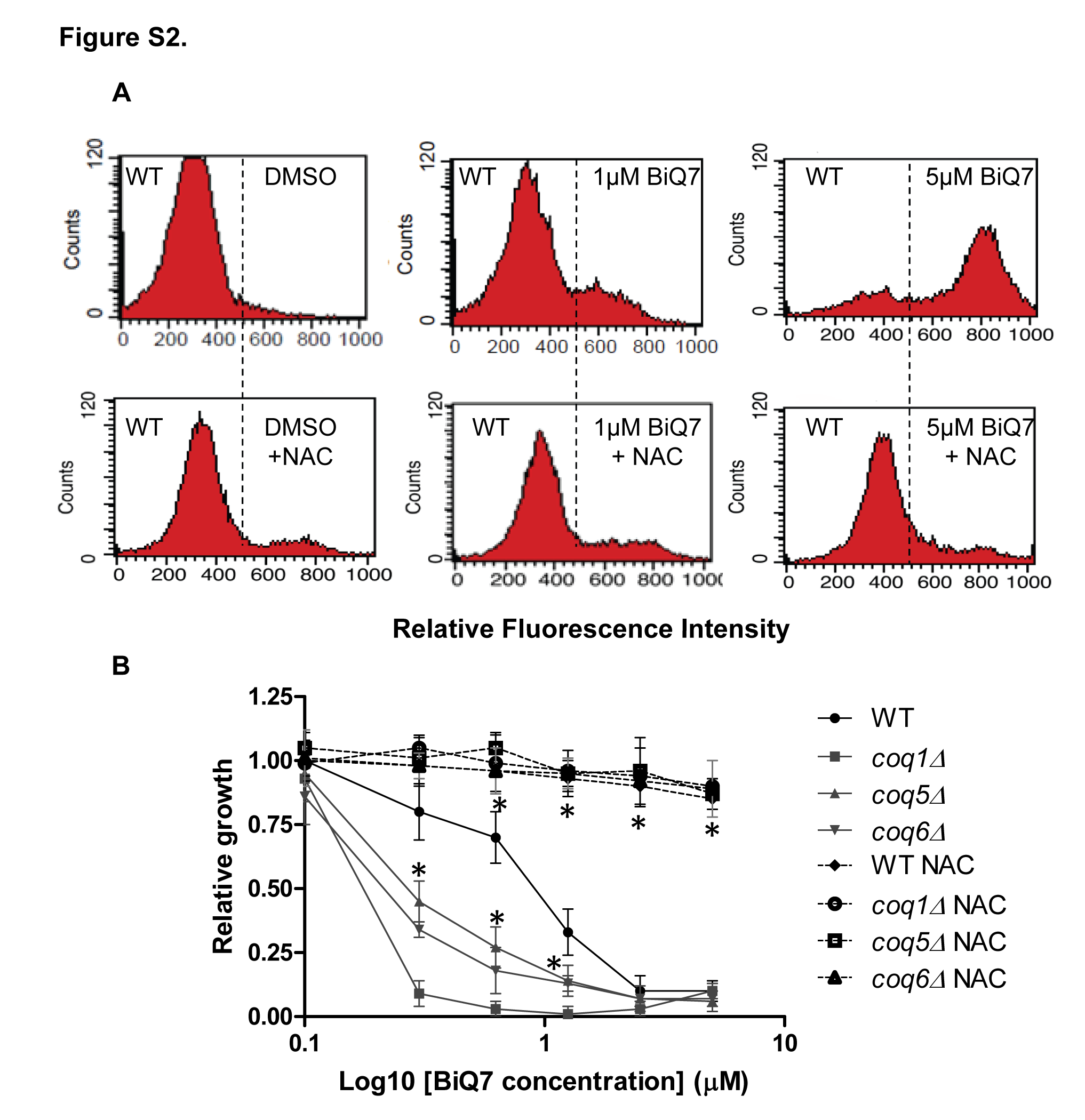

Supplement: Figure S2 — Neutralization of BiQ free radical generation and cytotoxicity by treatment with N-acetylcysteine (NAC). (A) Abrogation of BiQ dependent ROS generation by coincubation with NAC. Yeast wild type strain was treated with BiQ7 at increasing concentrations in the presence or absence of NAC for 2h and then incubated with DHR 123 for 1h. Experiments were performed three times with similar results. (B) Suppression of yeast growth by binaphthoquinone is neutralized by addition of NAC. Decreased growth of wild type and sensitive yeast mutants was rescued by addition of 100 µg/mL of NAC. Log-phase cultures at an OD600 of 0.1 were treated with different concentrations of BiQ7 or DMSO over 24h. Relative growth of wild-type and mutant strains in the presence of BiQ7 was measured against that of the yeast grown in the presence of DMSO. Growth curves were performed in triplicate and represent the average of three experiments. *p<0.05 for differences between wild-type and NDE1 over-expressing or mutant yeast. (0.58 MB TIF) [file pone.0010846.s002.tif]

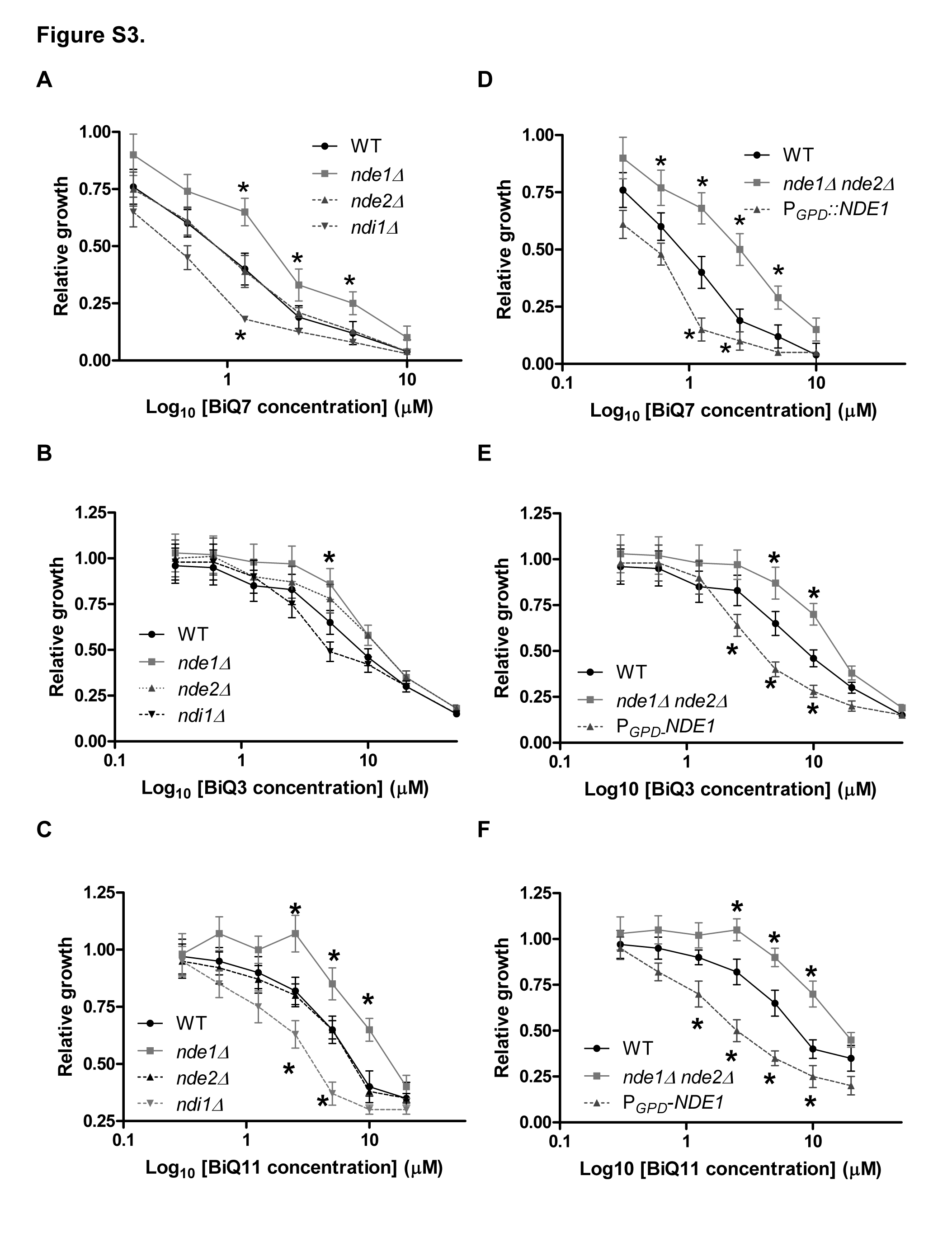

Supplement: Figure S3 — Deletion of nde1 nde2 enhances resistance to BiQs while overexpression of NDE1 enhances sensitivity to BiQs. (A,D) BiQ7 (B,E) BiQ3, (C,F) BiQ11. Log-phase cultures at an OD600 of 0.1 were treated with different concentrations of corresponding binaphthoquinones or DMSO over 24h. Relative growth of wild-type and mutant strains in the presence of binaphthoquinones was measured against that of the yeast grown in the presence of DMSO. Growth curves were performed in triplicate and represent the average of three experiments. *p<0.05 for differences between wild-type and mutant yeast. (0.39 MB TIF) [file pone.0010846.s003.tif]
